# Renin and the IGFII/M6P Receptor System in Cardiac Biology

**DOI:** 10.1155/2013/260298

**Published:** 2013-10-27

**Authors:** Jacqueline Heger, Klaus-Dieter Schlüter

**Affiliations:** Physiologisches Institut, Justus-Liebig-Universität Gießen, 35392 Gießen, Germany

## Abstract

Nonenzymatic cardiac activities of renin are well described during the last years and contribute either to cardiac-specific effects of the renin-angiotensin-aldosterone-system (RAAS) or to the pharmacological effects of RAAS inhibition. The interaction of renin with insulin-like growth factor II/mannose-6-phosphate (IGFII/M6P) receptors participates in nonclassical renin effects and contributes to cardiac remodelling caused by RAAS activation. The current findings suggest an important role for renin IGFII/M6P receptor interaction in cardiac adaptation to stress and support the idea that excessive accumulation of renin during inhibition of RAAS directly contributes to blood pressure-independent effects of these pharmacological interventions. It becomes a challenge for future studies focussing on chronic hypertension or myocardial infarction to comprise regulatory adaptations of the kidney, the main source of plasma renin and prorenin, because they directly contribute to key steps in regulation of cardiac (mal)adaptation via IGFII/M6P receptors. This receptor system is part of peptide/receptor interactions that modifies and possibly limits adverse remodelling effects caused by angiotensin II. Evaluation of interactions of renin with other pro-hypertrophic agonists is required to decide whether this receptor may become a target of pharmacological intervention.

## 1. Introduction

The renin-angiotensin-aldosterone system (RAAS) has an outstanding position in cardiac adaptations that balance blood pressure and body requirement in response to orthostase reaction or physical stress. Although a quick and effective increase in blood pressure is required to withstand the challenge to physical stress, uncontrolled activation of this system leads to chronic hypertension. High blood pressure is a major risk factor for adverse cardiac events such as stroke, myocardial infarction, and chronic heart disease. Therefore RAAS which plays a major role in many types of chronic hypertension is a main target for antihypertensive treatment regimes. However, it has well been recognized that RAAS is a complex network of biologically active peptides and their corresponding receptors that go far beyond the proper control of blood pressure. It is also clear that not all side effects of peptides participating in this system and the activation of their corresponding receptors are necessarily inducing adverse effects on cardiac tissues. A nice example for this is the different role of angiotensin II type one and type two receptors in intracellular signalling (as reviewed in detail in [1]).

In recent years it has been noticed that the first step in the RAAS cascade, the release of renin from juxtaglomerular cells in response to an activation of the sympathetic nervous system, is far more than the release of an aspartyl protease required for converting angiotensinogen into angiotensin I. This review will focus on those effects of renin that are mediated by stimulation of cardiac-specific insulin-like growth factor II/mannose-6-phosphate receptors (IGFII/M6P) and will summarize our current understanding of how renin expression and posttranslational modification will lead to activation of this receptor. The central question is how this will influence the adaptation to chronic pressure overload and cardiac stress in general. Although we are far from a complete understanding of these basic questions, there are already enough data supporting the idea that renin-dependent IGFII/M6P receptor activation participates in structural remodelling of cardiomyocytes. Furthermore, it has anti-hypertrophic properties as well. Thereby it potentially counteracts an angiotensin II-dependent adverse remodelling.

## 2. The Role of Renin in the Renin-Angiotensin-Aldosterone System (RAAS) in Cardiac Adaptation to Pressure Overload 

**Figure 1:**
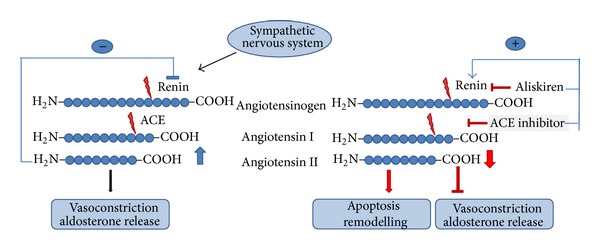
Coupling of sympathetic nervous system (SNS) to the renin-angiotensin system. Activation of the SNS increases the release of renin that forms angiotensin I from angiotensinogen. Angiotensin I is further converted to angiotensin II by angiotensin-converting enzyme (ACE). Angiotensin II is the effector molecule triggering the known effects of angiotensin II aimed to increase blood pressure. Via its inhibition on renin release it also possesses a feedback loop (left side). Inhibition of the RAAS by either ACE inhibition, AT receptor antagonism, or direct renin inhibition increases the release of renin but lowers the concentration of angiotensin II. Thereby, it reduces AT receptor-dependent effects but exerts new effects linked to remodelling (right side).

RAAS is one of the major systems involved in proper blood pressure control and it is causally involved in various cardiac-specific adaptations of the heart either to chronic pressure overload or to the consequences of myocardial infarction. In general two steps of proteolytic activation contribute to the effect. The first step converts angiotensinogen to angiotensin I and the second step converts angiotensin I into angiotensin II. Angiotensin II is considered as the most important molecule of this pathway. Renin, released from juxtaglomerular cells, causes the proteolytic cleavage of angiotensinogen to angiotensin I which is also a target of proteolytic cleavage. Angiotensin-converting enzyme (ACE) cleaves angiotensin 1 into angiotensin II. Finally angiotensin II acts on angiotensin receptors of which two types have been described, named type one and type two. Type 1 receptors are G-protein coupled receptors triggering most of the well described effects of angiotensin II on cardiac and vascular cells whereas type 2 receptors seem to be less prevalent and antagonize the action of type 1 receptors. There are important extensions to this system that have been described in greater detail before (see [2] for details). First, various site products such as angiotensin IV can be formed specifically in the presence of ACE inhibition or angiotensin receptor blockade and they activate mas receptors. Second, inhibition of angiotensin II by ACE blockade can be bypassed by chymases. Third, in addition to the classical angiotensin II-dependent effects on natrium retention, blood pressure, induction of thirst, and others, angiotensin II also acts on the release mechanisms of renin, thereby forming a feedback inhibition of the system (Figure 1). As a consequence of this any pharmacological inhibition within this cascade, such as ACE inhibition or receptor blockade, attenuates this feedback inhibition leading to an enhanced release of renin and increased plasma renin concentration (Table 1). It has been speculated that this increased renin release may hamper the beneficial effect of RAAS inhibition [3]. This has been a matter of debate since the direct renin inhibitor aliskiren may increase renin plasma concentration more effectively than ACE or AT1 receptor inhibition [4].

However, it may also be true that renin acts in nonenzymatic ways and improves cardiac remodelling and function and that the increase in renin plasma concentration offers the possibility that renin participates in anti-remodelling and anti-hypertrophic effects. Such nonenzymatic effects may be generated by binding to and activation of IGFII/M6P receptors. Here we discuss possible pathways by which such effects may be performed. These effects of renin require mannose-6-phosphate transfer to renin and prorenin but no aspartyl proteolytic activity of renin. In other words, antagonizing parts of RAAS either by direct inhibition of renin activity by aliskiren, by inhibition of ACE, or by angiotensin receptor blockade enhances plasma levels of renin and prorenin. This increases the ability of both molecules to activate IGFII/M6P receptors. Last but not least, the enzymatic activity of renin can be enhanced by binding to and activating prorenin receptors in cardiac tissues.

## 3. Renin: Transcription, Translation, and Regulation of Its Release

An extended review about the molecular understanding of the regulation of renin transcription, translation, and release has recently been published in a review article covering the physiology of kidney renin [11]. We will address only those topics of this area that are of specific interest for the role of renin in cardiac effects triggered by IGFII/M6P receptors. The renin gene was mapped to chromosome I and represents a protein with high interspecies homology, that is, 73% sequence homology between sheep and human renin, 85% sequence homology between rat and mouse DNA, and 68% sequence homology between rat and human renin [12–15]. Its 5′ flanking region has a classical promoter function and the transcriptional regulation is further modified by a renal enhancer. The promoter region covers 123 bp from −117 to +6 (positions relative to the transcriptional start site). It contains a classical TATA box [16]. A row of important cis-regulatory elements is located downstream of −200 which are also important in transcriptional regulation [17]. Renin is a cAMP-inducible gene that contains a classical cAMP response element (CRE) in the promoters of its human and mouse gene. Subtle distinctions may be seen with respect to differences in the response between these species [18–20]. In addition, the renin gene is also sensitive to PPAR*γ* [2] which may be of particular interest in the context of cardiac effects of renin as some angiotensin receptor antagonists have PPAR*γ* activating properties [21]. PPAR*γ* activation downregulates angiotensin type 1 receptor expression but enhances renin expression. Thus the effect of PPAR*γ* activation is rather complex but again directs RAAS to nonclassical plasma renin effects via IGFII/M6P receptors [22]. However PPAR*γ* is less relevant in rodents in this context [23]. During embryonic development, HOX and NOTCH pathways control the expression of renin [24]. In addition to its tight transcriptional control renin mRNA can also be stabilized, that is, by Y-Box protein-1 (YB-1), or destabilized, that is, by hydroxyacyl-CoA-dehydrogenase/3 ketoacyl-CoA thiolase/enoyl CoA hydratase beta subunit (HADHB) [25, 26]. The steady state mRNA expression of renin is increased by activation of the cAMP/PKA pathway via two different mechanisms: (a) an increase of its transcription and (b) stabilizing its mRNA. It is suppressed by activation of the PKC/calcium pathway that reduces the expression of renin and destabilizes its mRNA. In principle, catecholamines increase renin expression (and release, see later), whereas angiotensin II, activating phospholipase C (PLC), suppresses its expression and forms a feedback inhibition [27]. It is generally accepted that the majority of circulating renin originates from the kidney (mainly from juxtaglomerular cells). Renin is translated into protein in juxtaglomerular cells as a preprorenin protein. After cleavage of its presequence, it is directed into the Golgi apparatus. Here renin undergoes posttranslational modification and this step seems to be required for renin trafficking into dense-core secretory vesicles from which renin can be released by regulated exocytosis [28]. Moreover, this glycosylation is also a prerequisite for renin uptake from circulation in other tissues such as the heart [29, 30]. Renin is glycosylated like a typical lysosomal protein. In a first step N-acetylglucosamine-1-phosphate is transferred to selected mannose residues on lysosomal proteins. This reaction is catalyzed by UDP-GlcNAc : lysosomal enzyme N-acetylglucosaminylphosphotransferase [31, 32]. N-acetylglucosamine is removed in a second step leading to the typical Man-6-P monoester signal required for binding to IGFII/M6P receptors. Nowadays, it is well accepted that any cardiac stress will also interfere with renal physiology, leading to the cardiorenal syndrome. It will be necessary to investigate the expression and regulation of the enzymes involved in M6P labelling of renin in future. Without these N-glycosylation sites, renin cannot be packed into dense-core secretory granules for regulated exocytosis and will be released in a rather constitutive way. Renin can be released as prorenin and renin. Although prorenin has no enzymatic activity it cannot be considered as an inactive molecule because it interferes with IGFII/M6P receptors due to its glycosylation and thus it can be activated by prorenin receptors [6, 29, 33]. The analysis of the exact mechanism by which renin secretion is controlled is hampered by the fact that renin-producing cell lines as well as cultured juxtaglomerular cells secrete renin in a constitutive way. In culture the cells lose the ability to direct renin into secretory granules [11]. In situ approximately 75% of all renin is released as prorenin in a constitutive form, whereas renin is released by only 25% in a regulated manner [34]. The extent of prorenin release by the constitutive pathway simply depends on the regulation of renin transcription (see above). If necessary, that is, under conditions of chronic salt depletion, the amount of renin release is increased by altering the number of renin-producing cells. The regulation of renin secretion is performed by changes of the membrane potential of juxtaglomerular cells. Angiotensin II depolarizes these cells and it suppresses renin release whereas cAMP-dependent pathways hyperpolarize these cells and increase their release [35, 36]. Pharmacological inhibition of the Na^+^, K^+^, 2Cl^−^ cotransporter activity by furosemide leads to hyperpolarization and release of renin [37]. Interestingly, myocardial infarction increases the renal expression of this cotransporter that results in depolarization of renin-secreting cells and reduction of renin release [38]. This already suggests renal side effects caused by myocardial infarction leading to dysregulation of renin release. This interaction requires future attention. Inhibition of renin release also depends on intracellular calcium levels. Plasma free calcium concentrations directly affect renin release probably by inhibition of calcium-dependent adenylyl cyclase isoforms [39, 40]. Furthermore, the cells also express calcium sensing receptors [41]. Stimulation of calcium sensing receptors by calcimimetics inhibits renin secretion [42]. In addition intracellular calcium is the trigger of angiotensin II-dependent feedback inhibition on renin release [43]. As expected, any interference with angiotensin II activity (ACE inhibition, direct renin inhibition, or angiotensin receptor inhibition) blocks this feedback and increases plasma levels of renin thereby increasing nonenzymatic effects of renin that depend on interaction with IGFII/M6P receptors.

## 4. IGFII/M6P Receptors: Structure and Regulation of Their Expression

It was one of the main advantages in the field during the last decade that renin was identified as a molecule that directly acts on cells of target tissues such as the heart. Receptors known as the (pro)renin receptor activate inactive prorenin, enhance renin activity, and generate receptor signalling [33]. Another receptor, IGFII/M6P receptor, is less specific for (pro)renin because it recognizes proteins marked by mannose-6-phosphate. As outlined above renin fits these criteria. There is evidence for the existence of another pathway that is able to internalize nonglycosylated renin [30]. Yet the physiological relevance of this finding requires further work. Concerning binding to IGFII/M6P receptors it is noteworthy that renin has three different N-glycosylation sites by which the protein can be marked with M6P. Upon this M6P attachment, it is able to bind to and activate the IGFII/M6P receptor. This receptor may either internalize renin or trigger direct cell-specific effects (see below). Considering these aspects the IGFII/M6P receptor is central for cardiac nonenzymatic effects of renin. The structure and the biochemical aspects of this receptor have been worked out well. In principle, the IGFII/M6P receptor is a protein with 15 repeated segments of 124 to 192 amino acids in its extracellular part, a short 23 amino acid residue transmembrane domain, and a 167 amino acid residue cytoplasmic domain. Approximately 5–10% of the whole protein is located at the cell surface and is able to interact with ligands that cover M6P moiety such as renin, latent TGF*β*_1_, thyroglobulin, proliferin, leukemia inhibitory factor, and granzyme B. The receptor can also bind molecules that are not marked by M6P such as IGF-II, retinoic acid, urokinase-type plasminogen activator receptor, and plasminogen.

**Figure 2:**
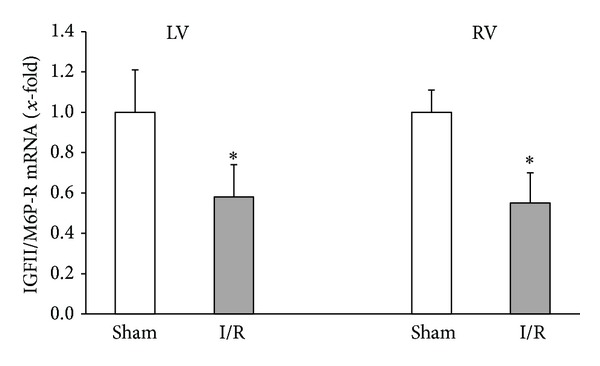
IGFII/M6P receptor mRNA expression in the left ventricle (LV) and right ventricle (RV) of rat hearts that underwent sham surgery or 30 min ischemia (ligation of the left arteria descendens) and one day of reperfusion (I/R). Data are means ± SD from *n* = 8 hearts. mRNA expression was quantified by real-time RT-PCR and expression is normalized to beta-2-macroglobulin (b2M). *P < 0.05 versus sham (unpublished observation).

In contrast to its biochemical characterization less is known about its expression, specifically in the heart and the regulation of receptor expression under stress conditions. It is nevertheless confirmed that IGFII/M6P receptors are highly expressed in the heart specifically in the developing myocardium [44]. Mice deficient in IGFII/M6P receptors die around the time of birth and show severe cardiac defects [45]. The most likely explanation is the loss of cleavage function of the receptor in these mice because IGFII and IGFI null backgrounds are rescued from perinatal lethality [46]. Cardiac-specific knockout of the IGFII/M6P receptor, however, is not lethal and has no obvious phenotype [47]. IGFII/M6P receptor expression mimics the expression profile of our “so-called” foetal genes in the heart, such as ANP, creatine kinase B, and myosin heavy chain *β*. As found for these molecules, IGFII/M6P receptor expression is reactivated in chronic pressure overloaded hearts [48]. As mentioned above, RAAS does not only participate in adaptation to chronic pressure overload but also to postinfarct remodelling. Preliminary experiments from our lab investigated the cardiac mRNA expression of the IGFII/M6P receptor in response to ischemia and reperfusion. In these unpublished experiments we found a downregulation of the receptor 1 day postinfarction in rats exposed to 30 min occlusion of the left anterior descending (LAD) coronary artery and subsequent reperfusion (Figure 2). Surprisingly this was found in the left and right ventricles indicating a hormonal regulation of IGFII/M6P receptor mRNA expression rather than a direct consequence of ischemia and/or reperfusion. Little is known about the regulation of IGFII/M6P receptors in cardiomyocytes but preliminary data on myoblasts may give some indication that angiotensin II upregulates the receptor expression [48]. But it is unclear whether these findings correctly reflect the regulation of IGFII/M6P receptor expression in cardiomyocytes. Since angiotensin II downregulates renin expression and release (see above), it is unclear why the receptor expression should be increased. In conclusion the analysis of IGFII/M6P receptor expression and its regulation requires future attention that has to be properly addressed.

## 5. IGFII/M6P Receptors in Cardiac Tissue: Specific Role and Comparison to Their Role in Other Tissues

**Figure 3:**
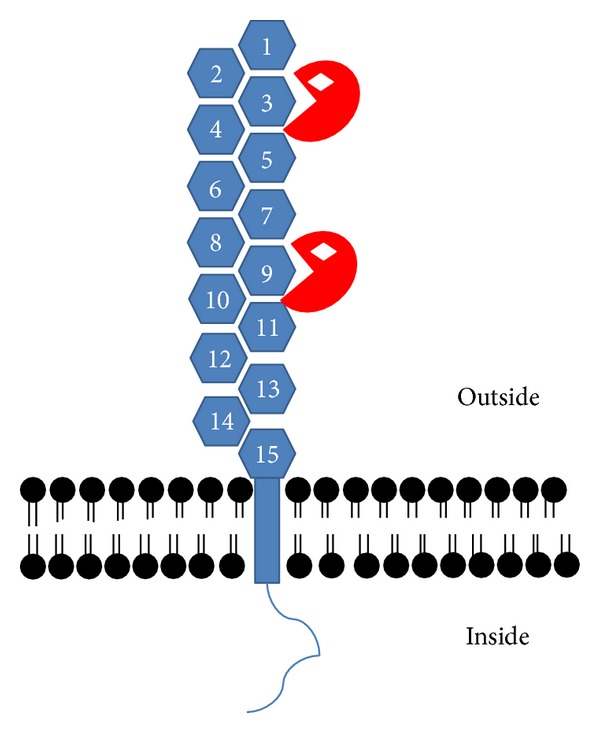
Structure of the IGFII/M6P receptor. The receptor has a cytoplasmic tail that triggers receptor internalization and signalling, a small transmembrane domain, and a large extracellular domain with 15 repeating segments. Mannose-6-phosphate labelled ligands (red symbol with white box for mannose-6-phosphate) bind to repeating segments 3 and 9. Unlike this schematic overview the most likely appearance of this receptor in living cells is that of a dimer.

The various physiological functions of the IGFII/M6P receptor can be ascribed to the numerous ligands that bind to this receptor (see above). The receptor is organized in a way that it covers a large extracellular domain with 15 repeated segments allowing binding of different types of ligands (Figure 3). In addition, the structure of this receptor enables it to function in two ways: as a clearance receptor through endocytosis in order to process or degrade proteins and as a signalling receptor that is attributed to G-protein dependent signals [49]. Most of the data so far cover the former role of IGFII/M6P receptors. One well studied ligand is IGFII. Unlike renin, IGFII is not labelled with mannose-6-phosphate and it interacts with the receptor via M6P-independent mechanisms. It binds at repeated segment 11, whereas mannose-6-phosphate labelled ligands bind at repeated segments 3 and 9. After binding to the M6P/IGFII receptor, IGFII becomes internalized, transported to lysosomes, and degraded, whereas the receptor is recycled back to the membrane. In this way the IGFII/M6P receptor regulates the extracellular level of IGFII and thereby controls the availability of IGFII [50]. An important role of this receptor function for cardiomyocytes was already suggested by the work of Kiess et al. [51] who could show in L6 myoblasts that IGFII in the medium of cultured cells is degraded largely by an IGFII/M6P receptor-mediated process. Nevertheless, this finding was never confirmed with cell culture models that represent more directly a fully differentiated type of cardiomyocytes.

Interestingly, cardiomyocytes express high levels of IGFII/M6P receptors, and already during embryonic development, the heart has the highest expression of the receptor of any foetal tissue [45]. Since the high receptor levels in foetal tissues decline in most tissues in late gestation and/or in the early postnatal period [52], the IGFII/M6P receptor seems to have a role in controlling normal foetal growth and development. Investigations with IGFII/M6P receptor knockout mice proved these assumptions. Lack of IGFII/M6P receptor resulted in foetal overgrowth and perinatal lethality of the transgenic progeny [45]. Further examination of the IGFII/M6P receptor knockout embryos yielded a 4-fold higher heart weight in the mutants than in wild type embryos at day 18.5. Perinatal lethality seems to be a result of cardiac abnormalities, including cardiac enlargement (38% mural thickening of the left ventricle), dilatation (89% increase), and septal and valvular defects [46]. Overgrowth of the ventricular myocardium is thereby not associated with cardiomyocytes hypertrophy but with hyperplasia due to an increase in cell number.

Moreover, Lau et al. [45] measured elevated levels of circulating IGFII- and IGF-binding proteins in mice lacking the IGFII/M6P receptor. Downregulation of IGFII/M6P receptor led to a decrease in internalization of IGFII and endocytosis in neonatal cardiomyocytes [53]. These data suggest that increased IGFII stimulates cardiac growth by alternatively binding IGFII to the IGFI receptor in such IGFII/M6P receptor knockout models. It remains to be elucidated whether cardiac downregulation of IGFII/M6P receptors as found in the early response to myocardial infarction is sufficient to reduce IGFII degradation and whether the same mechanism participates to the regulation of tissue-specific renin levels.

M6P/IGFII receptor-facilitated endocytosis is not restricted to IGFII. Work on cells distinct from cardiomyocytes suggests that IGFII/M6P receptors may also trigger cardiac growth responses modified by retinoic acid. In mouse macrophages overexpressing the IGFII/M6P receptor binding of retinoid acid induced growth inhibition and reduced spreading of these cells, which could not be seen in mouse macrophages lacking the IGFII/M6P receptor. So, IGFII/M6P receptor functions in mediating the growth-retarding effects of retinoids. Comparable effects of the retinoid acid have been seen in cultured neonatal rat cardiomyocytes [54].

Aside from IGFII and retinoid acid, neonatal cardiomyocytes as well as cardiac fibroblasts are able to bind and internalize recombinant renin and prorenin via M6P/IGFII receptors. Endocytosis of the prorenin/receptor complex results in intracellular activation of prorenin [29]. It was also shown that neonatal cardiomyocytes are capable of binding and activating native human prorenin of renal origin like plasma of probands with renal artery stenosis or plasma of hypertensive patients treated with captopril. However, native prorenin binds to a lesser extent than recombinant prorenin or renin possibly because the presence of growth factors in human body fluids leads to receptor dephosphorylation resulting in decreased M6P/IGFII receptor internalization [55].

Besides its well documented role as a “clearance receptor,” the IGFII/M6P receptor can also function as a signalling receptor. In neonatal cardiomyocytes interaction of the M6P/IGFII receptor with G*α*q results in activation of PLC*β* and calcineurin and leads to apoptosis [56]. Chen et al. [53] showed that IGFII/M6P receptor downregulation resulted in decreased sensitivity of cardiomyocytes to hypoxia- and TNF*α*-induced apoptosis. They explained these results with an improper trafficking and activation of cathepsins. It has been shown that these proteases can be bound, transported, and activated by the IGFII/M6P receptor [7]. Cathepsins are also known to induce apoptosis by TNF*α* in HeLa human epithelial carcinoma cells [57] or by oxidative stress in neonatal cardiomyocytes [58]. In neonatal rat ventricular cardiomyocytes IGFII induces apoptosis when IGFI receptor was downregulated. In this case the authors used IGFI receptor short hairpin RNA to downregulate the receptor [56]. Likewise, selective activation of IGFII/M6P receptor by ^Leu27^IGFII results in apoptosis by activating the intrinsic apoptosis pathway. M6P/IGFII receptors are known to be involved in the activation of other precursor proteins carrying the M6P recognition site like procathepsin, other aspartyl proteases, or latent TGF*β* [7, 59]. Some of them have been shown to trigger apoptosis in cardiomyocytes [60, 61]. In summary these data suggest the participation of IGFII/M6P receptors in proapoptotic events but the available data on cardiac cells are limited to neonatal cardiomyocytes. Any conclusion for the role in cardiac remodelling of the adult heart remains speculative unless a verification of such findings has been performed in adult cardiomyocytes and whole tissue preparations. As a controversial example, data of trophoblast cells with IGFII/M6P receptor stimulation may be taken. In these cells receptor activation resulted in decreased apoptosis possibly due to a different receptor G-protein coupling. In trophoblast cells IGFII-induced migration is mediated by IGFII/M6P receptor coupled to G*α*i that leads to an inhibition of adenylate cyclase A [62].

The role of IGFII/M6P receptor activation in the context of cardiac hypertrophy is not fully understood. Specific activation of these receptors leads to cardiac hypertrophy in a G-protein-dependent pathway in H9c2 cardiomyoblast cells. Downstream effectors are PKC*α* and CaMKII resulting in upregulation of ANP and BNP [63]. Interestingly, our studies with cultured adult ventricular cardiomyocytes showed that renin stimulates the elongation of cardiomyocytes [64]. This effect of renin is clearly dependent on IGFII/M6P receptor activation because elongation of cardiomyocytes could be antagonized by M6P but not by glucose-6-phosphate. It requires an activation of ERK1/2 consistent with findings in HEK cells as well [65]. The renin effect on elongation was antagonized by activation of PPAR*γ*, another hormone that has been introduced as a factor mediating the nonclassical renin effects (see above). These experiments in rats indicate that renin and PPAR*γ* regulate the length of cardiomyocytes. As explained before, PPAR*γ* may be of less relevance in rodents. Therefore mammals other than rodents have to be analyzed additionally to clarify this interaction in the light of clinical relevance of heart failure and remodelling. Nevertheless, renin is the first hormone identified so far to affect cell length rather than cell thickness as has been shown for all other pro-hypertrophic agonists. Of note, the effect of renin on cell shape of cardiomyocytes does not seem to be a cell culture phenomenon as it could be reproduced with cardiomyocytes isolated from transgenic rats (TGR (mRen2) 27). Future studies will have to analyze the interplay between renin and classical pro-hypertrophic agonists, such as angiotensin II, endothelin 1, and catecholamines on cardiac growth. A key question addressed so far is to define cellular mechanisms that may explain elongation of cardiomyocytes. Thereby the effect of increasing cell size by prolongation of contractile subunits (sarcomeres) in series compared to an increase in cell size by cell thickening that means positioning of sarcomeres in parallel on cardiac function needs to be analyzed. This is the cellular counterpart of concentric and eccentric hypertrophy and is not clarified on the cellular level.

In conclusion, all data so far show that IGFII/M6P receptors and their agonist renin are involved in cardiac remodelling. Increased cardiac IGFII/M6P receptor expression, found in patients with end-stage heart failure, underlines the relevance of this receptor system during the development of heart failure [66]. Yet, we still have to define the role of this system in this process in greater detail.

## 6. Interaction between TGF***β*_**1**_** and IGFII/M6P Receptor Stimulation

TGF*β*_1_ is a cytokine that regulates cardiomyocytes apoptosis [61]. It is upregulated in the transition from compensated hypertrophy to heart failure [67]. TGF*β*_1_ is embedded into RAAS because its cardiac expression is induced by angiotensin II [68]. TGF*β* is synthesized as an inactive precursor molecule that consists of the TGF*β* dimer, latency-associated proteins (LAPs), and latent TGF*β* binding protein (LTBP) [69]. To obtain mature TGF*β* this complex must be further processed. It is important in the light of this review that IGFII/M6P receptors play an important role in activating TGF*β*. About two decades ago it has been shown that latent TGF*β* (LTGF*β*) can bind to IGFII/M6P receptor at M6P recognition sites with either recombinant LTGF*β* [70] or LTGF*β* isolated from platelets [71]. In face of this review it is essential that the IGFII/M6P receptor is required for the activation of latent TGF*β* [59].

The physiological relevance of IGFII/M6P receptor in activation of latent TGF*β* has been analyzed in several studies. Using a receptor inhibitor, PXS25, human proximal tubule (HK-2) cells exposed to high glucose released less amounts of active TGF*β*. Also, hyperglycemia-induced increase in matrix proteins was inhibited by preincubation with PXS25. As the production of extracellular matrix proteins in this system depends on TGF*β* activation these data demonstrate IGFII/M6P receptor's participation [72]. It is likely to assume that similar mechanism also participates in the induction of cardiac fibrosis. The activation of latent TGF*β* by IGFII/M6P receptors may be more complicated and possibly requires the involvement of plasmin. Cell culture experiments showed that migration of bovine aorta endothelial cells can be severely impaired by bovine smooth muscle cells and fibroblasts [55]. Cell-cell contacts induce the activation of latent TGF*β* via plasmin since inhibitors of plasmin prevent this migration inhibition. Using antibodies against IGFII/M6P receptor migration of endothelial cells did not occur demonstrating that IGFII/M6P receptor is required for activation of TGF*β* [59]. Another pathway for the activation of LTGF*β* also involves the fibrinolytic system. IGFII/M6P receptor binds urokinase-type plasminogen activator receptor (uPA-R) and modulates its subcellular distribution [73]. In human monocytes the membrane contains a large complex consisting of IGFII/M6P receptor and uPA-R [74] that can additionally bind LTGF*β* and plasminogen. A proposed mechanism is the binding of plasminogen to IGFII/M6P receptor that is converted to active plasmin by uPA-R leading to activation of LTGF*β*. In HUVECs (human umbilical-vein endothelial cells) the association of IGFII/M6P receptor and uPA-R is essential for the activation of LTGF*β*, release of TGF*β*, and apoptosis induction [46]. Inflammation induced the generation of miniplasminogen (a proteolytic fragment of plasminogen) that then binds to IGFII/M6P receptor which associates with uPA-R. This binding results in formation of the active protease miniplasmin and activation of LTGF*β*. Interestingly, human umbilical-vein smooth muscle cells cannot activate LTGF*β*. This is due to the fact that IGFII/M6P receptor and uPA-R are not colocalized in these cells. Whether similar mechanisms occur in cardiomyocytes and contribute to the tissue-specific formation of active TGF*β* remains to be evaluated. In summary, an activation of RAAS induces TGF*β*_1_ expression and release and the activation of the cytokine requires IGFII/M6P receptor interaction. Once activated, TGF*β* induces apoptosis and fibrosis. On the other hand, inhibition of RAAS will decrease TGF*β* release and increase plasma renin levels favouring renin-dependent effects of this receptor. Thus, IGFII/M6P receptors are required for renin-dependent and renin-independent effects in cardiac remodelling.

## 7. Conclusive Remarks

Renin is far more than an aspartyl protease. Via binding to IGFII/M6P receptors it directly participates in the process of cardiac adaptation to cardiac stress such as pressure overload or myocardial infarction. These effects of renin require specific attention as they are intensified under conditions of pharmacological blockade of RAAS. Cardiac renin-dependent effects seem to be part of beneficial effects evoked by RAAS inhibition independent of the reduction of afterload. However, IGFII/M6P receptors are not specific for renin and the interaction between the effects of different ligands of this receptor system remains to be clarified.

## Figures and Tables

**Table 1:** Selected examples of the effect of pharmacological intervention at various sites of the RAAS on plasma renin concentration.

Species	Site of inhibition	Renin concentration		References
		Basal	Treatment	
Human	Renin (aliskiren)	14.9 mU/L	48.4 mU/L	[5]
Human	ACE (ramipril)	14.4 mU/L	35.2 mU/L	[5]
Human	AT1 (irbesartan)	17 mU/L	56 mU/L	[6]
Human	Renin (aliskiren)	5.6 pg/mL	34.9 pg/mL*	[7]
Human	AT1 (irbesartan)	5.6 pg/mL	11.3 pg/mL	[7]
Rat (SHR)	Renin (aliskiren)	9.3 ± 0.9 mg/kg	11.3 ± 1.9 mg/kg	[8]
Rat (SHR)	ACE (captopril)	9.3 ± 0.9 mg/kg	87.1 ± 61.0 mg/kg*	[8]
Rat (SHR)	ACE (captopril)	6.3 ± 1.6 ng/mL	27.4 ± 6.7 ng/mL*	[9]
Rat (SHR)	AT1 (irbesartan)	9.3 ± 0.9 mg/kg	143.0 ± 33.5 mg/kg*	[8]
Mouse (ApoE^−/−^)	Renin (aliskiren)	755 ± 90 ng/mL	3760 ± 567 mg/mL	[10]
Mouse (ApoE^−/−^)	AT1 (irbesartan)	755 ng/mL	11235 ± 3001 ng/mL	[10]
